# Parents’ experiences and perceptions of the acceptability of a whole-hospital, pro-active electronic pediatric early warning system (the DETECT study): A qualitative interview study

**DOI:** 10.3389/fped.2022.954738

**Published:** 2022-08-30

**Authors:** Holly Saron, Bernie Carter, Sarah Siner, Jennifer Preston, Matthew Peak, Fulya Mehta, Steven Lane, Caroline Lambert, Dawn Jones, Hannah Hughes, Jane Harris, Leah Evans, Sarah Dee, Chin-Kien Eyton-Chong, Enitan D. Carrol, Gerri Sefton

**Affiliations:** ^1^Faculty of Health, Social Care and Medicine, Edge Hill University, Ormskirk, United Kingdom; ^2^Clinical Research Division, Alder Hey Children’s NHS Foundation Trust, Liverpool, United Kingdom; ^3^Department of Women’s and Children’s Health, Institute of Life Course and Medical Sciences, University of Liverpool, Liverpool, United Kingdom; ^4^NIHR Alder Hey Clinical Research Facility, Alder Hey Children’s NHS Foundation Trust, Liverpool, United Kingdom; ^5^Department of General Paediatrics, Alder Hey Children’s NHS Foundation Trust, Liverpool, United Kingdom; ^6^Institute of Translational Medicine, University of Liverpool, Liverpool, United Kingdom; ^7^Institute of Infection, Veterinary and Ecological Sciences, University of Liverpool, Liverpool, United Kingdom; ^8^Department of Infectious Diseases, Alder Hey Children’s NHS Foundation Trust, Liverpool, United Kingdom; ^9^Oncology Unit, Alder Hey Children’s NHS Foundation Trust, Liverpool, United Kingdom; ^10^Faculty of Health, Public Health Institute, Liverpool John Moores University, Liverpool, United Kingdom; ^11^High Dependency Unit, Alder Hey Children’s NHS Foundation Trust, Liverpool, United Kingdom; ^12^Paediatric Intensive Care Unit, Alder Hey Children’s NHS Foundation Trust, Liverpool, United Kingdom

**Keywords:** pediatric early warning system (PEWS), parents experience, acceptability, clinical deterioration alert, qualitative

## Abstract

**Background:**

Failure to recognize and respond to clinical deterioration in a timely and effective manner is an urgent safety concern, driving the need for early identification systems to be embedded in the care of children in hospital. Pediatric early warning systems (PEWS) or PEW scores alert health professionals (HPs) to signs of deterioration, trigger a review and escalate care as needed. PEW scoring allows HPs to record a child’s vital signs and other key data including parent concern.

**Aim:**

This study aimed to explore the experiences and perceptions of parents about the acceptability of a newly implemented electronic surveillance system (the DETECT surveillance system), and factors that influenced acceptability and their awareness around signs of clinical deterioration and raising concern.

**Methods:**

Descriptive, qualitative semi-structured telephone interviews were undertaken with parents of children who had experienced a critical deterioration event (CDE) (*n* = 19) and parents of those who had not experienced a CDE (non-CDE parents) (*n* = 17). Data were collected between February 2020 and February 2021.

**Results:**

Qualitative data were analyzed using generic thematic analysis. Analysis revealed an overarching theme of trust as a key factor that underpinned all aspects of children’s vital signs being recorded and monitored. The main themes reflect three domains of parents’ trust: trust in themselves, trust in the HPs, and trust in the technology.

**Conclusion:**

Parents’ experiences and perceptions of the acceptability of a whole-hospital, pro-active electronic pediatric early warning system (The DETECT system) were positive; they found it acceptable and welcomed the use of new technology to support the care of their child.

## Introduction

Regular assessment, monitoring and recording of a child’s vital signs are key components in the surveillance of a child’s condition, fundamental to early detection of clinical deterioration, and core aspects of high-quality nursing and medical care (1–3). Outcomes of unrecognized clinical deterioration are a source of harm (4), and may result in a longer hospital stay, unplanned admissions to intensive care units (ICU) or high dependency units (HDU), further deteriorations, cardiac arrest or death (2, 5–8). However, there is good evidence that many children who die or deteriorate unexpectedly in hospital have observable features in the period before the seriousness of their condition is recognized (1, 2, 6, 7, 9). Failure to recognize and respond to clinical deterioration in a timely and effective manner is an urgent safety concern, driving the need for early identification systems to be embedded in the care of children in hospital (7, 10).

Pediatric early warning systems (PEWS) (4, 11, 12) or PEW scores (13–15) are a means by which health professionals (HPs) can be alerted to signs of deterioration, trigger a review and escalate care as needed. PEW scoring allows HPs to record a child’s vital signs and other key data including parent concern (16, 17). A PEW score is calculated either manually or electronically with each component valued according to its variance from normal (18); the overall PEW score indicates the risk of deterioration and prompts for action from HPs (19). PEW scoring can be recorded either on paper or an electronic system (4, 19, 20) although evidence suggests electronic-based scoring has benefits (accuracy and efficiency) over paper-based scoring (21, 22). Within the United Kingdom a “plethora of practices, tools and initiatives” (4) exists. There is conflicting evidence of the effectiveness of PEWS and PEW scores (10, 23), reflecting the complexity of implementing scores and systems, what outcomes are being measured, and the complexity of evaluating their implementation.

Having or being a child who is unwell and who is hospitalized for any reason and for any duration of time, can be an emotional, uncertain time for any child or parent (24). The impact of clinical deterioration requiring escalation of care and unplanned transfer to intensive care reaches beyond the child’s physical condition and affects parents psychosocially and emotionally. Parents whose child requires admission to an intensive care unit have their lives turned upside down and liken the experience to “riding a rollercoaster” (25). Some parents (e.g., those with children with complex medical conditions) may be “experts” in their child’s vital signs and health status and may recognize changes before HPs (26). However, this is not always the case and only a few studies have examined parents’ understandings of vital sign observations or experiences of PEW systems (16, 17) and it is noted that few PEWS include parent concern as an item (27) with calls for parents’ concerns to be investigated in future studies (28). This lack of attention to parents’ knowledge is perhaps surprising considering they are potentially well placed to raise concerns about changes in their child’s condition before deterioration and changes in vital signs happen.

This paper reports findings from a sub-study of the Dynamic Electronic Tracking and Escalation to reduce critical Care Transfers (DETECT) study (22). The DETECT study implemented a proactive end-to-end deterioration solution (the DETECT surveillance system) across a tertiary children’s hospital. The DETECT surveillance system is supported by System C’s CareFlow Connect and Vitals (pediatric version) apps. These apps were modified for the study and are known as DETECT e-PEWS. DETECT e-PEWS is an electronic observation and decision support system which is uploaded onto iPods, iPads or personal devices approved by Trust Information Governance, and used by HPs. Once vital signs are recorded onto the iPod at the child’s bedside, an age-specific PEW score is automatically calculated, and the PEW score provides instant bedside decision support. In the case of scores reflecting higher risk of deterioration, this prompts an escalating series of actions such as increasing frequency of monitoring, requesting a medical review or activation of the resuscitation team. The iPods and iPads communicate across the hospital’s electronic information system and enable recorded data to be visible in real-time. The algorithms underpinning the scores, alerts and triggers and actions to be taken were bespoke for this study. Definitions of the DETECT-related terms used in the paper can be found in Table 1.

**TABLE 1:** Definitions of DETECT-related terms.

Term	Definition
The DETECT Study	Dynamic Electronic Tracking and Escalation to reduce Critical care Transfers (DETECT): a stepped wedge mixed method study to explore the clinical effectiveness, clinical utility and cost-effectiveness of an electronic physiological surveillance system for use in children.
DETECT (surveillance) system	A proactive end-to-end deterioration solution implemented across a tertiary children’s hospital with the aim of screening children for early signs of serious deterioration or sepsis and reducing complications and emergency transfers to critical care following deterioration in hospital.
DETECT e-PEWS	The DETECT surveillance system is supported by System C’s CareFlow Connect and Vitals (pediatric version) apps. These apps were modified for the study and are known as DETECT e-PEWS. DETECT e-PEWS is used by health professionals to document vital signs on iPods and escalate concern and to respond to alerts of deterioration triggered by the system using iPods, iPads or by personal mobile device.

In the sub-study presented in this paper we used semi-structured telephone interviews with parents of children admitted to the study hospital where DETECT e-PEWS was being used to explore its acceptability. We define acceptability according to the Theoretical Framework of Acceptability’s (TFA) (v2) (29) definition as a *“multi-faceted construct that reflects the extent to which people delivering or receiving a healthcare intervention consider it to be appropriate, based on anticipated or experienced cognitive and emotional responses to the intervention*. The TFA encompasses seven constructs: (1) affective attitude, (2) burden, (3) ethicality, (4) intervention coherence, (5) opportunity costs, (6) perceived effectiveness, and (7) self-efficacy.

The main research question was: What are the experiences and perceptions of parents about the acceptability of newly implemented electronic surveillance system and what factors influence acceptability? A sub-question was: Are parents aware of signs indicating clinical deterioration and do they feel able to raise related concerns with HPs?

## Materials and methods

### Study design

An interpretive description qualitative, semi-structured telephone interview design. This manuscript is reported according to Consolidated Criteria for Reporting Qualitative Research (COREQ) guidelines.

### Participants and setting

Parents of children who were in-patients (excluding children admitted as day-cases or to the pediatric intensive care unit and neonatal surgical unit) at Alder Hey Children’s Hospital, a pediatric tertiary setting in Liverpool, United Kingdom were invited to participate in semi-structured telephone interviews. Recruitment occurred between February 2020 and February 2021. Two groups of parents were recruited: those whose child had experienced a critical deterioration event (CDE) during their admission (CDE parents, *n* = 19) and those whose child had not (non-CDE parents, *n* = 17). A CDE was defined as a deterioration where the patient is critically unwell, which culminates in an emergency transfer to high dependency unit or the intensive care unit, or an unexpected death. Families of children who died were not included. Convenience sampling was used to recruit participants from the two groups.

Dedicated DETECT study research nurses approached parents to explain the interview study and to provide tailored information sheets. Parents who showed an interest gave permission for their contact details to be shared with the researcher, a female academic, with experience in interviewing children, young people and families and who would be conducting the interviews with them (HS). The initial contact by the researcher was by text message, as this was felt to be the least intrusive approach as many of the parents were known to be present on wards or units within the hospital. Following contact by text message, parents were then telephoned at a time agreed to be convenient to them, consent was gained, and the interview undertaken (HS).

### Semi-structured telephone interviews

Data were collected *via* semi-structured telephone interviews based on an interview schedule (15 questions) for both the CDE and non-CDE parents. The questions asked parents about their child and the reason for their admission, their experience of their child having their vital signs (their “observations” often referred to as “obs”) assessed and recorded, their experience of DETECT e-PEWS in use, and their perceptions of the acceptability and functionality of DETECT e-PEWS as well as any challenges or improvements they could suggest. Questions were also asked that related to parental concern about clinical deterioration, such as whether they can tell if their child is “getting poorlier” (deteriorating) and whether they felt able to raise a concern. All telephone interviews were audio-recorded, and duration ranged from 20 to 40 minutes. Transcripts were not returned to participants for comments or corrections, and no repeat interviews were carried out. Data saturation appeared to be achieved when no new perspectives were being added and the datasets seemed complete enough to achieve the aims.

### Parent involvement and engagement

Parents were involved in the development of the DETECT study, contributed to the design of the information sheets and informed the development of the interview schedule ensuring that our approach and questions were sensitive to parents’ feelings.

### Ethics

This study gained ethics approval *via* the North West, Liverpool East Research Ethics Committee (IRAS ID: 215339). All those involved in gaining consent were suitably qualified, experienced, and trained and consent was gained in accordance with the principles of Good Clinical Practice on Taking Consent (30). All relevant governance protocols relating to data management and anonymization were followed.

### Analysis

The semi-structured telephone interviews were analyzed (HS, BC) using the six stages of thematic analysis (31, 32). The first stage, “familiarization” began with a thorough read through of each of the transcripts to acquire an overview of the data. The next stage, “generating initial codes” involved reading the transcripts with the intention of finding the natural meaning of parents’ expressions and accounts; this also involved coding and collating interesting and relevant data into potential themes. In the “reviewing themes” stage, the themes were checked to see if they worked across the data set. In the final two stages, themes were “defined and named” and key extracts and quotations drawn upon in producing and “writing up” the findings/report. Quotations appearing in the text are linked to participant numbers and whether they were a CDE or non-CDE parent (e.g., P27, CDE). Software was not used to code or analyze the data and participants did not provide feedback on the findings.

## Results

Thirty-six parents (*n* = 33 mothers, *n* = 3 fathers; *n* = 19 CDE parents, *n* = 17 non-CDE parents) participated in the telephone interviews (see Table 2).

**TABLE 2:** Parent and child demographics from parent interview responses.

	Experienced a critical deterioration event (CDE)	Not experienced a critical deterioration event (non-CDE)
Parent status	N (19)	N (17)
Mother	18	15
Father	1	2
**Child Gender**		
Girl	6	12
Boy	13	5

**FIGURE 1:**
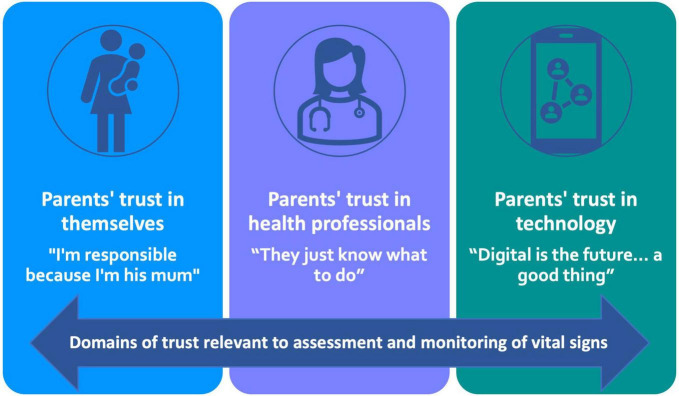
Main themes showing domains of trust.

Our initial attention had been on the parents’ perceptions of acceptability and their experiences of their child having their vital signs recorded and monitored by the electronic surveillance system. However, our qualitative analysis revealed an overarching theme of trust. Trust was a key factor that underpinned all aspects of their child’s vital signs being recorded and monitored. The main themes reflect three domains of parents’ trust: trust in themselves, trust in the HPs, and trust in the technology (see Figure 1).

The three themes and associated sub-themes (Table 3) are discussed with anonymized excerpts from the interviews.

**TABLE 3:** Main themes and sub-themes.

Theme 1: Parents’ trust in themselves when their child is unwell	
Sub-themes	• Feeling responsible but glad to share the load
	• Being knowledgeable about what to look for, but not relying on the technology
	• Knowing enough but sometimes needing more information
**Theme 2: Parents’ trust in health professionals when their child is unwell**	

Sub-themes	• Feeling safe “in their hands” yet confident to raise a concern
	• Relationships were not impacted by technology
	• Noticing the “gadgets”
**Theme 3: Parents’ trust in technology when their child is unwell**	

Sub-themes	• Digital is the way forward
	• A better system that minimizes error

### Trust

Although recording vital signs may be seen as a routine aspect of care, it is an essential component of monitoring a child’s condition. Both CDE and non-CDE parents trusted HPs to monitor their child’s vital signs and respond as needed; trust is a component of acceptability. Although our focus was on DETECT e-PEWS and the DETECT surveillance system, it was clear that acceptability of these factors for parents was not considered in isolation but within the wider context of experiencing their child being unwell and hospitalized. One of the parents commented that the use of the iPods *“didn’t really change anything*…*it didn’t make much difference to us as the parents”* (P13, non-CDE); perhaps revealing a sense that when a system is functioning effectively, the components that contribute to it working are largely invisible.

### Parents’ trust in themselves when their child is unwell

Parents’ trust in themselves provides important context for the other themes as it is characterized by parents’ ongoing sense of responsibility to be with and look after their child, their ability to recognize the signs that their child is becoming more unwell or getting better (at home and in hospital), and their need for more information.

#### Feeling responsible but glad to share the load

Remaining responsible for their child during their in-patient stay was described as “*just part of being a parent*” (P17, non-CDE) or seen as something that was self-evident, “*I’m still his mum*” (P3, non-CDE) regardless of location “*[whether] I’m in hospital or at home”* (P11, non-CDE). Parents perceived that:

“[HPs are] there to treat her medically, I’m there to care for her…I’m her first caregiver, no matter where she is” (P16, non-CDE).

Parents were the constant in their child’s care during their admission. This constancy was evident in the way they talked of being with their child up to *“24 h a day”* (P24, CDE) because *“I don’t like leaving my child”* (P20, CDE). It reflected a deep-seated need to be with their child, especially when their child had not started to recover, with one mother saying that *“I wouldn’t have left his side if I didn’t feel like he was getting better”* (P27, CDE). Their constant presence meant parents felt that they were able to “*see the early signs [of deterioration]”* (P22, CDE) and change more easily and quickly than HPs because the HPs are only *“coming in and checking and stuff but I’m with him all the time”* (P11, non-CDE).

None of the parents interviewed thought it was solely the responsibility of the HPs to look after and out for their child. The parents suggested that responsibility for assessing their child was a shared role between themselves and the HPs as:

“the nurse looks out for them, but as a parent, you know when they’re getting poorly…so it’s joint really” (P23, CDE).

#### Being knowledgeable about what to look for, but not relying on the technology

Parents were knowledgeable about what vital signs were being recorded with most parents able to say how often vital signs were recorded, such as *“every hour on HDU but every 2 or 3 h on the ward”* (P23, CDE) and what was recorded, including but not limited to *“oxygen saturation”* (P25, CDE), *“heart rate”* (P24, CDE), “*blood pressure*” (P1, non-CDE) and *“temperature*” (P26, CDE).

Additionally, parents were aware of the subtle signs of change in their child’s health, outside of the typical vital signs that they knew HPs recorded. Both the parents of CDE and non-CDE children looked out for similar signs and behaviors and emphasized the importance of their role in identifying them. Parents judged whether their child’s condition was improving or deteriorating by physical cues such as *“looking at her color”* (P20,CDE) “*how hard he was working, how much his chest was sucking in, how much his little neck was going in”* (P26, CDE), how “*he was grunting, like a pain noise”* (P27, CDE) and when their child “*wasn’t eating*… *and she wasn’t putting weight on so was dropping off the centiles”* (P2, non-CDE). They also considered personality traits such as how “*happy and cheeky”* (P7, non-CDE) their child was or changes in their child’s level of *“alertness”* (P23, CDE). One parent emphasized the important role parents play in looking out for subtle changes in their child’s health, outside of the recorded vital signs, especially when:

“[my child] doesn’t display typical [signs of deterioration], they can look very well but be very poorly” (P2, non-CDE).

Some parents drew on data from the technology and the machines in their child’s room to inform them when something had changed or was not right. Some parents said they knew what to look out for and that it was *“just a case of looking at the screen*” (P8, non-CDE) or paying attention to when the machine “*alarms when things [vital signs] are out [different from normal]”* (P25, CDE). These were indications that their child was getting worse. Another parent said they knew what was being recorded because “*it’s all on display on the monitors, it’s not like there’s any secrets because I could see it myself*” (P24, CDE). However, some parents stated that they did not always understand the “numbers” or what was displayed on the screens. One parent discussed they knew their child was becoming more unwell, not because of their own parental instincts or from the technology around them, but how they *“could tell from the doctors’ faces when they walked in the room*” (P27, CDE) and that this made them feel “*nervous, scared and anxious”* (P27, CDE).

#### Knowing enough but sometimes needing more information

Although most parents felt satisfied that *“everything was explained straight away”* (P18, CDE) because the nurses “*tell us exactly what they’re doing*…*they always tell you what is going on”* (P2, non-CDE), this was not the case for all parents. One parent commented that *“[HPs] would come in using it [the iPod] and then they would go”* (P24, CDE) and another said *“nurses just take the readings and just go*…*they just leave the room so we don’t know what’s going on”* (P19, CDE). One parent made it clear that *“what you don’t want is someone to come in, do something, not say anything and then go out”* (P2, non-CDE).

Some parents did not want detailed information but simply needed reassurance that their child was doing well; reassurance typically occurred after completion of vital signs such as *“they put it all in and then they’ll say to me ‘Oh he’s fine’*” (P22, CDE) and “*all fine, I’m watching her”* (P24, CDE). However, a few parents suggested that there were missed opportunities for communication from the HPs to explain their child’s vital signs and overall condition as “*no-one really tells you what’s written down*” (P24, CDE) or “*they weren’t explained*… *the results weren’t shared”* (P6, non-CDE).

### Parents’ trust in health professionals when their child is unwell

In this theme the focus shifts to the role of the HPs and the trust that parents have that they will care for their child and that the technology will help support that care and keep their child safe. Parents talked about their trust in the HPs, with some describing them as *“family”* (P35, CDE). This trust was particularly based on the bonds they built with HPs during their child’s hospitalization and how the relationships with them let them be parents to their child rather than carers; one parent explained:

“when you see a nurse step in and do it [care for your child] right and explain it to you, you can go back to parent mode and that’s what I needed to do, and they gave me the chance to do that” (P6, non-CDE).

One father whose child had a long-term condition, described the nurses as his child’s “*aunties and her sisters*” because the nurses had *“been [with] her, her whole life”* (P30, CDE). Another parent talking about the HPs caring for her child said they *“didn’t realize that there’s this whole big world of wonderfulness of being able to fix people*” (P21, CDE).

#### Feeling safe “in their hands” yet confident to raise a concern

Most of the parents interviewed spoke highly of the HPs involved in their child’s care, and some named individuals who had played a positive part during their child’s hospital admission. Parents talked of feeling both *“safe”* (P25, CDE) leaving their child *“in their hands*” (P25, CDE) but also feeling confident enough to raise concerns with HPs about any aspect of their child’s care including when “*things were getting missed and escalating*” (P6, non-CDE). Parents welcomed the “parent concern” question which HPs asked as part of the DETECT e-PEWS vital signs assessment. Most parents felt “*there was always someone to ask”* (P11, non-CDE) and that “*having it explained makes it all seem less scary*” (P5, non-CDE). Many recalled times when they had become worried about their child and had raised concerns and asked the HPs “*to have a look”* (P20, CDE) or *“come and check over”* (P23, CDE). Parents were clear that HPs *“always ask, are we concerned. and listened to us a lot”* (P33, CDE) and saw this as important information to enter along with *“the numbers”* (P33, CDE) into the iPods. One parent expressed a sense of reassurance in DETECT e-PEWS as it was likely to help them raise concerns about their child as:

“even if they [HP] don’t know, they’ll use the [iPod] and then flag up to a doctor and say ‘Right, we’ll get a review before it gets any worse”’ (P22, CDE).

Most of the parents could see that the use of the technology by the HPs, in conjunction with parental concern, were effective ways of escalating concerns and alerting more senior HPs. One parent discussed this saying, *“if he looks like he’s working a bit harder to breathe they’ll [nurses]*… *use that to flag up a doctor and say he may need reviewing”* (P22, CDE).

#### Relationships were not impacted by technology

Positive, trusting relationships between parents and professionals existed because of *“the way they [HPs] are with them [the children] and the way they are with us [the parents]”* (P22, CDE). Trust, rapport and good interaction between the HPs and the child were not negatively impacted by the using an iPod to record the child’s vital signs into DETECT e-PEWS and, as one parent noted, the HPs gave them *“their attention”* (P10, non-CDE) whilst using the iPod. One parent indicated that the iPod improved this interaction, stating *“even though they’re on the [iPod], they’ll still stand there and speak to him and say ‘Do you need anything*?”’ (P22, CDE) and they compared this to previous admissions which involved the HP “*going out [of the cubicle] to write it down*” (P22, CDE). Another parent suggested that HPs *“pay more attention to her [their child] whilst they are doing it [recording the vital signs]”* (P20, CDE) using the iPod. One parent commented that the “*only thing this changes*… *it’s more efficient*” (P27, CDE) emphasizing that the new technology was accepted and functioning as it should.

#### Noticing the “gadgets”

All the parents interviewed had seen HPs using the iPods, and most parents could recall multiple occasions when a nurse had used the iPod to record their child’s vital signs, but it was rare for a parent to recall noticing a doctor using either the iPods or iPads associated with the DETECT system. Most parents were able to describe what technology the DETECT e-PEWS used saying *“it’s like an iPod, isn’t it*?” (P22, CDE) or *“on those phone looking things”* (P20, CDE) and one parent commented that it was their child who first noticed the technology saying, “*the girls [nurses] have got iPods, mum”* (P3, non-CDE).

In cases where the parents had not been informed about the purpose of the iPods and DETECT e-PEWS, very few assumed that the HP was using their personal mobile phone. If they did, there was an assumption that the use was legitimate, for example, *“she’s counting on her phone, you know like the time or whatever”* (P29, CDE). One parent revealed inherent trust, saying that the HPs *“on the ward they are just that good you don’t even think about them being on the phone while they are there*” (P13, non-CDE). However, when parents had not been informed, it did make a few of them wonder what the professional was doing, “*it does look like they’re texting*… *I think it’s important you tell parents what it is otherwise it does look like they’re on their phones*” (P5, non-CDE). Other parents worked out what the iPods were for, explaining *“I’d kind of worked out what they were doing just by the fact that they were stood with their phone looking at the monitors”* (P28, CDE). Some parents who initially thought that the HPs were using their phones, took an active stance and asked them, *“what are these? [the iPods]”* (P35, CDE); one asked a student nurse as they knew they were *“very knowledgeable”* (P15, non-CDE).

Some parents whose children had experience of previous admissions, explained *“things had changed since the first time we were in, so I noticed that they’d got the little gadgets to do it on which I thought was good because it’s instantly in the system then, isn’t it?”* (P24, CDE). Most parents felt that the HPs’ attitudes to the iPods (and DETECT e-PEWS) were positive, noting they seem to “*like them*… *now they’ve got used to them”* (P35, CDE), with one parent saying some HPs “*have said it’s easier doing it this way [using the iPod]”* (P22, CDE). One parent commented that “*everyone seems quite competent in what they’re doing*” (P4, non-CDE).

### Parents’ trust in technology when their child is unwell

Every parent reported a good acceptance of technology playing a part in their child’s care. Two key reasons were proposed: belief that digital is the way forward, and positive attitudes and experiences relating to the functionality of the device/system.

#### Digital is the way forward

Many of the positive accounts demonstrated parents’ positive personal beliefs about technology. Some parents proposed that new technology, in general, was progressive and a good thing because *“it’s better to be paperless”* (P27, CDE), *“digital is the future, it’s an advancement”* (P24, CDE), and *“technology’s the way forward”* (P7, non-CDE). Parents were aware that the system was novel, with one parent noting *“there’s no kind of like [similar] system”* (P36, CDE). This openness to the implementation of new technology meant that parents were not hostile or unreceptive to the idea of technology being used. One parent presented a balanced view, having a positive outlook on the system but expressing a concern that digital information *“may be sent to the wrong place”* (P9, non-CDE).

#### A better system that minimizes error

Functionality is a key aspect of acceptability and although the parents trusted the HPs, they appreciated that the purpose of the system was to minimize errors or mistakes. One parent explained that using the iPod as part of DETECT e-PEWS helped reduce their own *“anxious [feelings]”* (P31-CDE) and agreed that the speed of *“nurses being alerted”* (P31, CDE) helped put her at ease. Another parent noted “*things are a bit quicker, a bit safer and things don’t go missing”* (P7, non-CDE). The notion of the system acting as a check was evident with some parents; one noted that:

“it checks that the observations are within the normal range for [my son] or any other child that’s in [hospital]. And it helps spot basically if they’re getting more poorly” (P12, non-CDE).

Reducing error and early alerts were deemed to be important aspects of functionality. One parent explained that DETECT e-PEWS and the DETECT system:

“takes away that human error side…and also gives you a catalog of the observations over a period of time so you can see if the patient is declining, you can see it ahead of time because there are early warning signs” (P14-non-CDE).

Others explained that it was good as *“it cuts down on mistakes*….*as it’s far less [open to] human error”* (P34, CDE) and that *“it’s all there ready*… *it alerts without having to go back through previous conversations”* (P7, non-CDE). One parent responded that they thought it was *“brilliant”* as there was *“less pressure on the nurses to get the information right*… *you can trust the information”* (P36, CDE) and that using technology meant there was a *“record they can trace”* (P19, CDE).

The systematic guidance when undertaking and recording vital signs was also seen as a positive component. One parent was reassured as DETECT e-PEWS meant HPs “*have a step-by-step guide*… *[on the iPod] so they won’t forget to do his temp [temperature] or forget to count his breaths, it’s all there for them to do”* (P22, CDE). Another parent felt that the real-time aspect could reduce delays, recognizing that using DETECT e-PEWS means that it *“notifies that someone’s deteriorating faster, and gets the right people looking at it, rather than them having to find a computer and*…*it taking like an hour”* (P23, CDE). Another parent explained that if their child needed more *“help and support, then the numbers are there for them [the HPs] to have a look and quickly see that she has gone up from this to this and know that’s not right”* (P20, CDE).

Acceptability was augmented for some parents when HPs had shared positive comments with them about DETECT e-PEWS and the DETECT system with one parent recalling that a nurse had said sometimes *“she’d take someone’s obs and get called to an emergency and you’d put them down and they’d go missing*… *she said they’re so much better”*… *[and]*… *“it’s just quicker for them and then once it’s done, it’s done”* (P27, CDE). One parent noted that the system:

“consolidates everything into one place…. I’ve not experienced that in any other hospitals, but it makes the treatment really fluid. I love it. I think it’s great” (P32, CDE).

## Discussion

This study aimed to explore the experiences and perceptions of CDE and non-CDE parents about the acceptability of a newly implemented electronic surveillance system (the DETECT surveillance system), and factors that influenced acceptability and their awareness around signs of clinical deterioration and raising concern. In summary, parents were open to and positive about the DETECT surveillance system; they found it acceptable and welcomed the use of new technology to support the care of their child.

**FIGURE 2:**
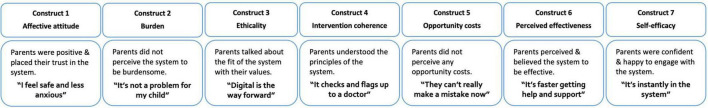
Domains of the Theoretical Framework of Acceptability (29) as applied to findings.

Parents’ experiences and perceptions of acceptability were positive across the seven TFA (29) constructs (C1-7), reflecting a range of factors related to but not solely about the electronic system (see Figure 2). These factors included the information they receive about DETECT e-PEWS, the system and the iPods, what it does, the relationship they have with HPs, their previous experiences and their role as a parent of a child requiring hospital care. The perceptions of acceptability did not differ between the CDE and non-CDE parents.

The “affective attitude” (TFA-C1) (29) the parents talked about most was trust based on a combination of confidence in their own knowledge, experience and abilities and that of the HPs along with belief in the benefits and functionality of the technology. Although the intention is not to provide a detailed examination of the concept of trust, it is important to note that typically trust is considered to be a multi-dimensional concept that is relational, dynamic and fragile, based on the benevolence, goodwill, and competence of another person and it is linked to notions of vulnerability (33–36). Parents also demonstrated trust and confidence in the “ethicality” (TFA-C3) (29) and value of digital technology and the devices; however, it was clear that these components were a small part of their experience of their child’s admission. Parents positioned the iPods (and DETECT e-PEWS and the DETECT system) within a bigger picture, and saw them as a very small but important part of their whole hospital experience in which multiple factors were at play. As with other parents of children in hospital, the parents in our study were focused on whether their child was getting better or worse, trusted that deterioration would be recognized and responded to (16, 37), and thus perceived the technology to be “effective” (TFA-C6) (29) by contributing to reducing risks (TFA-C6) (29) through prompting recognition and response. Parents reported very few “opportunity costs” (TFA-C5) (29) beyond wondering what would happen if the internet failed or if information was misplaced. Any such costs were small compared to the benefits they perceived as being inherent in the system (TFA-C6) (29). Parents understood the principles of the DETECT system demonstrating “intervention coherence” (TFA-C4) (29) and were happy to engage with the system (TFA-C7) (29) as they appreciated that DETECT e-PEWS ensured complete sets of vital signs were recorded, and immediate alerts were triggered.

The parents perceived their own role in assessing their child’s condition as being important and one that complemented that of the professionals. They recognized that their constant presence at their child’s bedside placed them in a particularly unique position to be a key part of the DETECT system. Parents have been described as the “go-between” for everyone involved in their child’s care, although it is recognized that this places increased responsibility on parents (38). This aligns with the parents in our study who talked of having both parental responsibility for, and the most complete account of, their child’s journey. These factors meant they considered themselves to be responsible as a key communicator of any changes they noted in their child’s condition. However, some research shows parents do not necessarily consider that their role includes alerting HPs about deterioration (39). None of the parents perceived the parental concern question which is part of DETECT e-PEWS as being problematic or a burden (TFA-C2) (29) and it was a clear way they could engage with the system (TFA-C7) (29); indeed, this was welcomed by them as they saw this as one of the ways HPs were keeping their child safe. It is clear from other research that parents value the opportunity to be heard and involved in their child’s care, particularly if they are experts in their child’s health care needs (26). Some research shows that parents are considered to be trustworthy partners in escalating care (39), and other studies show that raising awareness is key to success (16). The value of DETECT e-PEWS is that it does raise awareness through asking the “parent concern” question and stimulating conversation about the child’s vital signs. Along with other benefits, we note that DETECT e-PEWS is not a burden to parents, which again is positive in terms of acceptability (TFA-C2) (29).

The parents’ accounts further our understanding of the use of technology in a child’s care; they were enthusiastic about the implementation of the DETECT e-PEWS and the DETECT system suggesting a good fit with their values (TFA-C3) (29). Not only does handheld technology such as iPods reduce the burden and time constraints on HPs (19, 40), but it provides a complete and easily accessible record of events with benefits similar to those reported in other studies of the use of PEW scores and PEWS (41, 42). Our research highlights that recording vital signs using the handheld devices in real-time at the child’s bedside provides parents with a focused opportunity to express concerns and ask questions. Other parent escalation research highlights the importance of parents being able to voice concerns and have these responded to (16, 17, 26).

### Limitations

Parents of children who are unwell and in hospital have unique and different experiences; our data offers a brief insight into their perspectives and may not be representative of other parents in other hospitals or different clinical situations. There were many more mothers included in interviews (*n* = 33) than fathers (*n* = 3) so, fathers’ views are underrepresented. Some of interviews were conducted whilst the child was still unwell in hospital, and this may have influenced parent responses, for example, parents whose child is acutely ill and/or requiring critical care may have a different view to a parent whose child is unwell but recovering, because of heightened emotions and anxiety about the future.

## Conclusion

Overall both CDE and non-CDE parents’ experiences and perceptions of the acceptability of a whole-hospital, pro-active electronic pediatric early warning system (The DETECT system) were positive. Regardless of whether they were CDE or non-CDE the parents’ experiences and responses were broadly similar. The findings were considered in relation to the TFA’s seven constructs and although there were some suggestions for improvements to be made, these were to do with the explanations of the “obs” and communication from nurses and not necessarily to do with DETECT e-PEW system or its functioning. Parents were accepting of the system and saw it as an advancement to the assessment, monitoring and care their child receives. They were aware of the system, and what it is used for. They felt that HPs were competent and able to use the system to record their child’s vital signs, and that the system would create automated alerts, as needed. Parents trusted themselves to recognize subtle signs of change in their child’s health, and the trusting relationship they had with the HPs meant they felt able to ask questions and raise concerns.

## Data availability statement

The datasets presented in this article are not readily available because of limitations within the ethics approval but are available from the corresponding author on reasonable request. Requests to access the datasets should be directed to detectstudy@alderhey.nhs.uk.

## Ethics statement

The studies involving human participants were reviewed and approved by the North-West, Liverpool East Research Ethics Committee (IRAS ID: 215339). The patients/participants provided their written informed consent to participate in this study.

## Author contributions

BC, GS, EC, and MP: conceptualization. EC and GS: data curation. HS and BC: formal analysis and writing—original draft. GS, EC, BC, and MP: funding acquisition. BC, GS, EC, and MP: methodology. CL: project data administration. BC: visualization. HS, BC, GS, EC, JH, SS, DJ, LE, HH, SD, FM, C-KE-C, JP, MP, SL, and CL: writing, review and editing. All authors contributed to the article and approved the submitted version.
